# Circulating tumor DNA in early-stage breast cancer: personalized biomarkers for occult metastatic disease and risk of relapse?

**DOI:** 10.15252/emmm.201505332

**Published:** 2015-06-01

**Authors:** Taija M af Hällström, Maija Puhka, Olli Kallioniemi

**Affiliations:** Institute for Molecular Medicine FIMM, University of HelsinkiHelsinki, Finland

## Abstract

The availability of blood-based markers to predict response of a solid tumor to treatment, estimate patient prognosis and diagnose relapse well before clinical symptoms arise, is a long-standing hope in clinical oncology. Ideally, assays designed to provide such information should be inexpensive (at least in the foreseeable future), simple, and, of course, predictive of the clinical evolution of the disease. While early research focused on circulating glycosylated tumor-derived protein biomarkers, the focus is now rapidly shifting to new opportunities, such as circulating tumor cells, extracellular vesicles, micro-RNAs and cancer-derived cell-free DNA a.k.a. circulating tumor-derived DNA (ctDNA).

See also: **E Olsson *et al*** (August 2015)

In this issue of *EMBO Molecular Medicine*, Olsson *et al* (2015) provide exciting evidence for the value of ctDNA measurements in early-stage breast cancer. Based on their results, elevated plasma ctDNA levels preceded clinical detection of relapse in 86% of the patients with an average lead-time of 11 months. Admittedly, this is a small retrospective study of 20 patients that needs to be validated as well as expanded and extended, but it is nevertheless a promising proof-of-concept for prospective translational and clinical studies. The study also points to a number of methodological aspects that may need to be considered by investigators studying ctDNA in any type of cancer.

The authors studied 20 patients with primary breast cancer who underwent surgery and adjuvant chemo- or radiation therapy. The primary tumors were sequenced to identify patient-specific genetic alterations, followed by the design of specific digital droplet PCR (dd-PCR) assays to detect these alterations in plasma samples. The main goal was to investigate whether the patient-specific plasma ctDNA assay could detect disease relapse and occult metastatic disease before clinical evidence of the recurrence. Rising ctDNA levels predicted clinical evidence of metastasis with an average lead-time of 11 months. The assay had a 93% sensitivity and 100% specificity. Thus, a positive plasma ctDNA test was always eventually followed by clinical detection of metastasis. Additionally, high ctDNA levels before surgery predicted poor disease-free outcome and overall survival. This suggests that ctDNA levels could in the future define patients who need additional adjuvant therapies after primary surgery. Although this study was not powered to fully evaluate the predictive and prognostic value of the plasma ctDNA assay, it serves as a strong stimulus for additional and larger clinical follow-up studies.

Compared to previous studies, the Olsson *et al* (2015) study provides a very optimistic picture about the sensitivity of ctDNA assays in primary breast cancer. Many previous studies had focused on metastatic disease, where the ctDNA concentrations are much higher (Murtaza *et al*, 2013). In a large study across different cancer types, Bettegowda *et al* (2014) found detectable levels of plasma ctDNA in 49–78% of patients with localized tumors from breast, colon, pancreas, and gastroesophageal cancers as well as in 86–100% of patients with metastatic tumors. Overall, in previous studies, ctDNA has already been reported to be useful for quantitation of tumor burden in response to surgery, treatments or as a measure of overall survival in a number of malignances, such as colorectal (Leary *et al*, 2010), breast (Dawson *et al*, 2013), ovarian and lung cancer (Murtaza *et al*, 2013).

The Olsson *et al* (2015) study also features several technical and methodological improvements that may contribute to optimal sensitivity and specificity. Firstly, blood was sampled serially: prior to surgery and at approximately 3–8, 12, 24, and 36 months after the primary operation. This longitudinal sampling is a significant advantage as it enables systematic comparison of plasma ctDNA levels with subsequent clinical progression of the disease. Secondly, the choice of the methods applied for plasma DNA isolation may be critical. In this study, the authors used a commercial kit, which although not previously validated for this purpose, appears promising based on the results obtained. Thirdly, 0.5 ml plasma samples were used to isolate circulating DNA, which is a smaller volume compared to many previous studies and suggests outstanding sensitivity of the assay. Fourthly, the authors selected about 10 tumor-specific rearrangements from the WGS data for each patient and validated 4–6 of these as dd-PCR assays from the diagnostic plasma sample. The increasing evidence pointing to tumor clonal heterogeneity as well as evolution during tumor progression raises significant concerns about how well the genomic makeup of the primary tumor reflects all the subclones in the eventual metastatic disease. As a consequence, the authors selected their dd-PCR assays to target genomic rearrangements with varying allele frequencies. Thus, if one such subclone was selected for, and became the dominating clone after relapse or in the metastases, this would be more likely to be detectable in the plasma ctDNA assay with this strategy.

Assuming that the results of this study will be validated in larger clinical studies with standardized, diagnostic-grade assays, is the plasma ctDNA assay otherwise applicable in clinical routine? Many additional considerations are required to answer this question. Will the prognostic and/or predictive value of ctDNA assays, such as for the detection of minimal residual disease, actually lead to clinical improvements in therapy or patient survival? This will be critical to investigate and will obviously be also dependent on the available therapeutic options to treat such sub-clinical disease. Will the whole-genome sequencing of the primary tumor (and matching germline DNA), followed by tailored dd-PCR assays, become practically and economically feasible and will the results be obtainable in a timely fashion to help patients prospectively in clinical practice? The answer to all these questions in the near future will probably be “yes”, given the significant decrease in genome sequencing costs and opportunities for laboratory automation. Indeed, even unbiased whole-genome sequencing of plasma ctDNA appears feasible in the near future (Leary *et al*, 2012), given the progress of sequencing technology at low template levels, such as single cells (Wang *et al*, 2014). The advantage of unbiased plasma ctDNA sequencing would be to obtain an overview of the multiple subclones that may co-exist in a patient with advanced disease, although it will obviously not achieve the depth of coverage and cancer specificity of carefully designed targeted PCR assays. We also need to better understand how ctDNA levels correlate with short-term therapeutic effects (e.g., therapeutic effects associated with cell killing and increased release of ctDNA in the plasma) and with long-term impact due to the remaining tumor mass in the body. A sharper picture of the biology and kinetics of ctDNA release and turnover should expand the utility of circulating nucleic acids as tumor markers.

In conclusion, plasma ctDNA assays represent a promising, highly customized diagnostic technology that may help us transition toward truly personalized oncology, whereby it will become possible to monitor and consequently optimize the therapeutic impact of any surgical, radiotherapy, or drug treatment in real-time.
